# JAG1 is associated with the prognosis and metastasis in breast cancer

**DOI:** 10.1038/s41598-022-26241-8

**Published:** 2022-12-20

**Authors:** Xiaojuan Qiao, Buhuan Ma, Weiting Sun, Ning Zhang, Yang Liu, Lizhou Jia, Caixia Liu

**Affiliations:** 1 Department of Oncology, Affiliated Hospital of Inner Mongolia Medical University, No.1, Tongdao North Road, Hohhot City, 010050 Inner Mongolia China; 2 Inner Mongolia Medical University, Hohhot City, 010050 Inner Mongolia China; 3 Central Laboratory, Bayannur Hospital, Bayannur, 015000 Inner Mongolia China

**Keywords:** Cancer, Cell biology

## Abstract

Jagged canonical Notch ligand 1 (JAG1) regulates the progression of many cancers by the Notch signaling pathway, but its role in breast cancer (BC) remains unclear. In this research, JAG1 protein expression in BC tissues was detected by immunohistochemistry. The association between JAG1 and clinical significance was analyzed. The effect of JAG1 on malignant behaviors of BC cells was demonstrated by in vitro experiments. JAG1 expression in BC tissues was higher than that in para-carcinoma tissues. High JAG1 expression was significantly linked to advanced lymph node metastasis, distant metastasis, and the TNM stage. JAG1 was an independent prognostic factor for BC patients. JAG1 knockdown inhibited the proliferation, motility, migration, and invasion of BC cells, and weakened adhesion and penetration abilities to the blood–brain barrier, whereas JAG1 overexpression had the opposite effects. JAG1 has the potential to be a prognostic marker and therapeutic target for BC patients.

## Introduction

Breast cancer (BC) is the most common malignant tumor and the main cause of cancer death in women. There were 2.26 million new cases of BC worldwide in 2020, accounting for 11.7% of total cancer cases^1,2^. With the development of molecular diagnostics, BC treatment has gradually developed into individual therapies guided by the molecular type, and the therapeutic effect has been improved greatly^3^. However, some BC patients still die from disease progression or distant metastasis during treatment. It is urgent to explore novel carcinogenic mechanisms and potential therapeutic targets.

The Notch signaling pathway is a highly conserved signaling pathway for cell–cell communication in biological evolution, playing an important role in various physiological processes^4–6^. The notch signaling pathway can convert transcriptional repressor Core-binding factor-1 to the transcriptional activator, and then regulate transcription of target genes that encode transcriptional regulatory proteins, participating in cellular bioprocess^7–10^. Increasing evidence confirmed that the Notch signaling pathway plays a major driving role in the occurrence, development, and distant metastasis of BC^11–13^.

As one of the most active cell surface ligands in the Notch signaling pathway, Jagged canonical Notch ligand 1 (JAG1) is overexpressed in many cancer types with a close relationship to tumor biology^14–16^. Reedijk et al.^17^ found a dose-dependent relationship between JAG1 mRNA and the overall survival of BC patients, which has gradually attracted attention to the mechanism of JAG1 in BC. JAG1 expression was significantly increased in metastatic BC tissues (bone, liver, lung, and brain metastases) compared with localized non-metastatic BC, and a high positive rate of JAG1 was correlated with malignant and invasive characteristics of tumors, indicating the major driving role of JAG1 in BC metastasis^18^.

This study detected JAC1 protein expression in BC tissues, and analyzed the relationships between JAG1 and clinicopathological features as well as patient prognosis. Furthermore, we explored the effect of JAG1 on the malignant behaviors of BC cells, particularly crossing the blood–brain barrier (BBB). This study suggests that JAG1 is a potential therapeutic target for advanced or metastatic BC.

## Results

### BC tissues overexpress JAG1 protein

**Figure 1:**
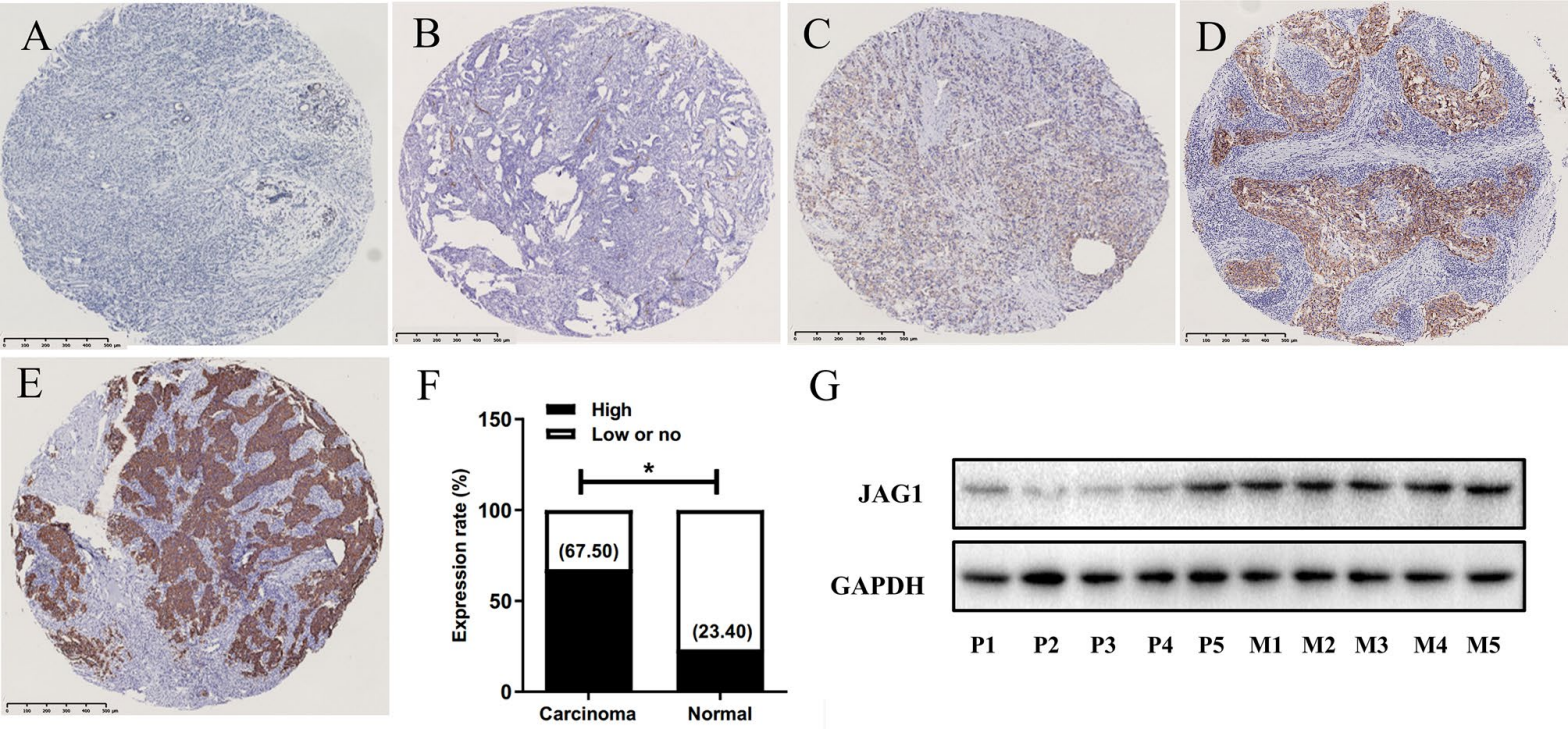
JAG1 protein expression in breast tissues. (**A**) Adjacent breast tissue with no JAG1 expression (IHC score, 0); (**B**) BC tissue with low JAG1 expression (IHC score, 40); (**C**) BC tissue with high JAG1 expression (IHC score, 100); (**D**) BC tissue with high JAG1 expression (IHC score, 160); (**E**) BC tissue with high JAG1 expression (IHC score, 240); (**F**) JAG1 protein expressions in breast tissues; (**G**) JAG1 expression in patients with or without brain metastases. P1–P5, patients without brain metastases. M1–M5, patients with brain metastases. *P < 0.05.

We detected JAG1 protein in 200 BC tissues and 47 para-cancer breast tissues by IHC. As shown in Fig. 1A–E, the JAG1 protein was mainly expressed in the cell membrane and cytoplasm of BC cells. The high expression rate of JAG1 in BC tissues was 67.5% (135/200), significantly higher than that in para-cancer breast tissues (23.4%, 11/47) (*P* < 0.001, Fig. 1F). In addition, we found the JAG1 expression in patients with brain metastases was higher than that in patients without brain metastases (Fig. 1G).

### JAG1 expression is associated with clinicopathological characteristics

To explore the influence of JAG1 on BC progression, we analyzed the relationship between JAG1 expression and pathological characteristics. The results confirmed that JAG1 was significantly associated with lymph node metastasis (χ^2^ = 15.034, *P* < 0.001), distant metastasis (χ^2^ = 11.148, *P* = 0.001), and the TNM stage (χ^2^ = 12.215, *P* = 0.002) (Table 1). However, JAG1 showed no association with menstruation, tumor location, tumor size, histological type, ER, PR, HER-2, TNBC, or Ki67.

**Table 1:** JAG1 protein expression level and BC patient clinicopathological characteristics.

Characteristics	n	JAG1 Expression		Pearson *χ*^2^	P-value
		Low or no	High		
Total	200	65 (32.50)	135 (67.50)		
**Menstruation**				0.961	0.161
Premenopausal	39	9 (23.08)	30 (76.92)		
Postmenopausal	161	56 (34.78)	105 (65.22)		
**Tumor location**				0.167	0.682
Left	121	38 (31.40)	83 (68.60)		
Right	79	27 (34.18)	52 (65.82)		
**Tumor size (cm)**				4.403	0.106
≤ 2	37	15 (40.54)	22 (59.46)		
> 2 and ≤ 5	156	50 (32.05)	106 (67.95)		
> 5	7	0 (0.00)	7 (100.00)		
**Histological type**				0.101	0.751
Invasive ductal	170	56 (32.94)	114 (65.06)		
Others^a^	30	9 (30.00)	21 (70.00)		
**ER**				2.538	0.111
Negative	110	41 (37.27)	69 (62.73)		
Positive	90	24 (26.67)	66 (73.33)		
**PR**				1.915	0.166
Negative	128	46 (35.94)	82 (64.06)		
Positive	72	19 (26.39)	53 (73.61)		
**HER-2**				2.580	0.108
Negative	113	42 (37.17)	71 (62.83)		
Positive	87	23 (26.44)	64 (73.56)		
**TNBC**				2.383	0.123
Negative	123	35 (28.46)	88 (71.54)		
Positive	77	30 (28.96)	47 (61.04)		
**Ki67**				0.079	0.778
Negative	11	4 (36.36)	7 (63.64)		
Positive	189	61 (32.28)	128 (67.72)		
**Lymph node metastasis**				15.034	< 0.001*
N0	70	35 (50.00)	35 (50.00)		
N1 + N2 + N3	130	30 (23.08)	100 (76.92)		
**Distant metastasis**				11.148	0.001*
M0	145	57 (39.31)	88 (60.70)		
M1	55	8 (14.55)	47 (85.45)		
**TNM stage**				11.990	0.001*
I	47	25 (53.19)	22 (46.81)		
II + III	153	40 (26.14)	113 (73.86)		

^a^Others include ductal in situ in 20 cases, papillary in 4 cases, and mucinous in 6 cases.

### High JAG1 expression is linked to a poor prognosis

**Figure 2:**
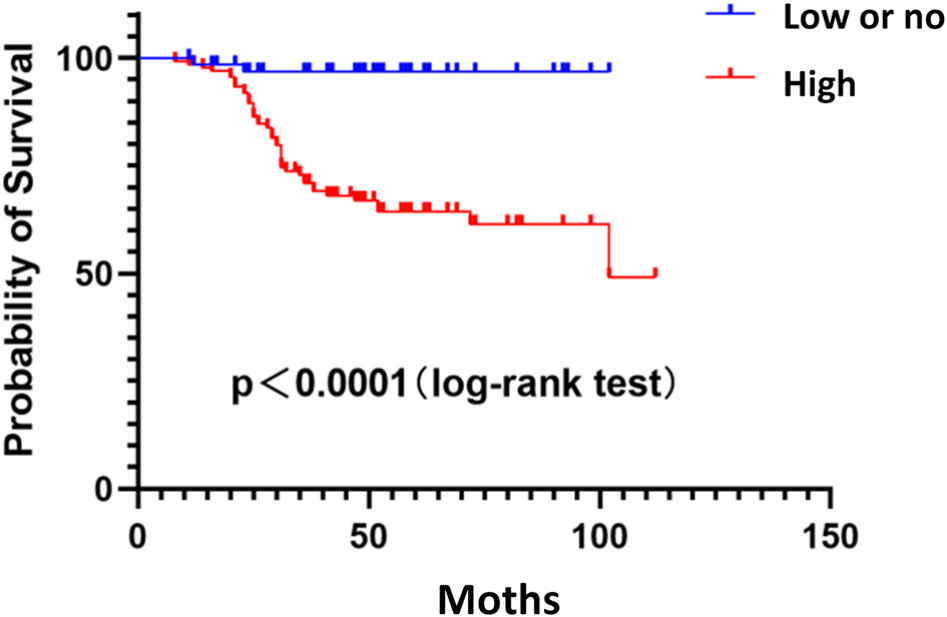
Survival curves of BC patients.

Univariate analysis demonstrated that risk factors for overall survival (OS) of BC patients included JAG1 expression (*P* < 0.001), the TNBC type (*P* = 0.031), lymph node metastasis (*P* = 0.001), distant metastasis (*P* < 0.001), and TNM stage (*P* < 0.001). Multivariate analysis confirmed that poor OS was significantly associated with high JAG1 expression (HR, 7.097; 95% CI: 1.671–30.146; *P* = 0.008) as well as TNBC (HR, 2.189; 95% CI: 1.209–3.962; *P* = 0.010), lymph node metastasis (HR, 4.396; 95% CI: 1.370–14.104; *P* = 0.013), distant metastasis (HR, 11.002; 95% CI: 4.880–24.803; *P* < 0.001), and the TNM stage (HR, 1.853; 95% CI: 1.178–2.719; *P* = 0.004) (Table 2). Kaplan–Meier survival analysis demonstrated that BC patients with high JAG1 expression showed poorer survival than other patients (Fig. 2).

**Table 2:** Univariate and multivariate analyses of the prognostic factors for overall survival in BC.

	Univariate analysis			Multivariate analysis		
	HR P -value 95%CI			HR P -value 95%CI		
**JAG1 expression**						
High vs low or no	12.028	< 0.001	2.914–49.651	7.097	0.008*	1.671–30.146
**Menstruation**						
Premenopausal vs postmenopausal	1.400	0.413	0.626–3.131			
**Tumor location**						
Right vs left	0.891	0.707	0.489–1.625			
**Tumor size (cm)**						
≤ 2 vs > 2 and ≤ 5 vs > 5	2.531	0.256	0.510–12.567			
**Histological type**						
Ductal in situ vs lnvasive ductal vs Papillary vs mucinous	1.201	0.400	0.784–1.838			
**ER**						
Negative vs positive	0.560	0.061	0.305–1.028			
**PR**						
Negative vs positive	0.541	0.068	0.280–1.046			
**Her-2**						
Negative vs positive	0.793	0.448	0.436–1.443			
**TNBC**						
Negative vs positive	1.901	0.031*	1.059–3.414	2.189	0.010*	1.209–3.962
**Ki67**						
Negative vs positive	1.154	0.843	0.279–4.767			
**Lymph node metastasis**						
N0 vs N1 + N2 + N3	5.853	0.001*	2.096–16.347	4.396	0.013*	1.370–14.104
**Distant metastasis**						
M0 vs M1	9.182	< 0.001*	4.802–17.558	11.002	< 0.001*	4.880–24.803
**TNM stage**						
I vs II vs III	2.317	< 0.001*	1.446–3.713	0.358	0.004*	1.178–2.719

HR, hazard ratio; CI, confidence interval.

*P < 0.05.

### Knockdown of JAG1 inhibits the malignant behaviors of MDA-MB-231 cells

**Figure 3:**
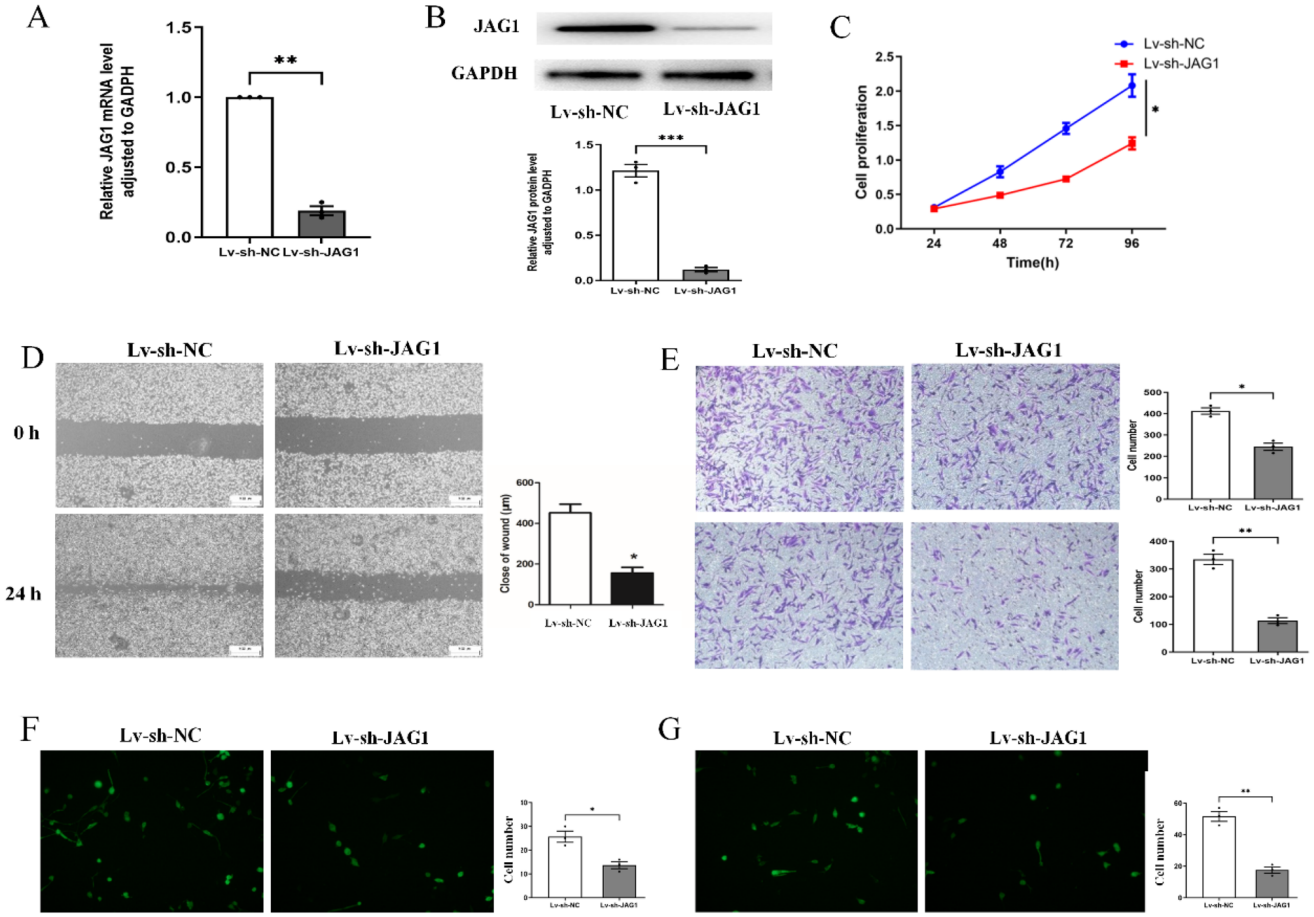
The influence of JAG1 knockdown on BC cells. (**A**) The JAG1 mRNA was evaluated by qPCR after JAG1 knockdown; (**B**) JAG1 protein was detected by Western Blot after JAG1 knockdown; (**C**) the proliferation of BC cells was detected by CCK-8 assay; (**D**) cell motility after JAG1 downregulation was detected by wound healing assay; (**E**) transwell assay was conducted to detect the migration (upper panels) and invasion (lower panels) of MDA-MB-231; BBB model was constructed to detect the adhesion (**F**) and penetration (**G**) abilities to BBB.

To further ascertain the function of JAG1, we downregulated JAG1 expression in the MDA-MB-231 (Fig. 3A, B). CCK-8 assays demonstrated that JAG1 knockdown significantly inhibited BC cell proliferation (Fig. 3C). A wound-healing assay confirmed that downregulation impaired the motility of MDA-MB-231 cells (Fig. 3D). Transwell assays revealed the inhibitory influence of JAG1 downregulation on migration (Fig. 3E, upper panels) and invasion (Fig. 3E, lower panels) of BC cells. Additionally, a BBB model revealed that JAG1 knockdown significantly weakened the tumor cell adhesion and penetration into the BBB (Fig. 3F, G).

### Overexpression of JAG1 promotes the malignant behaviors of MDA-MB-231 cells

**Figure 4:**
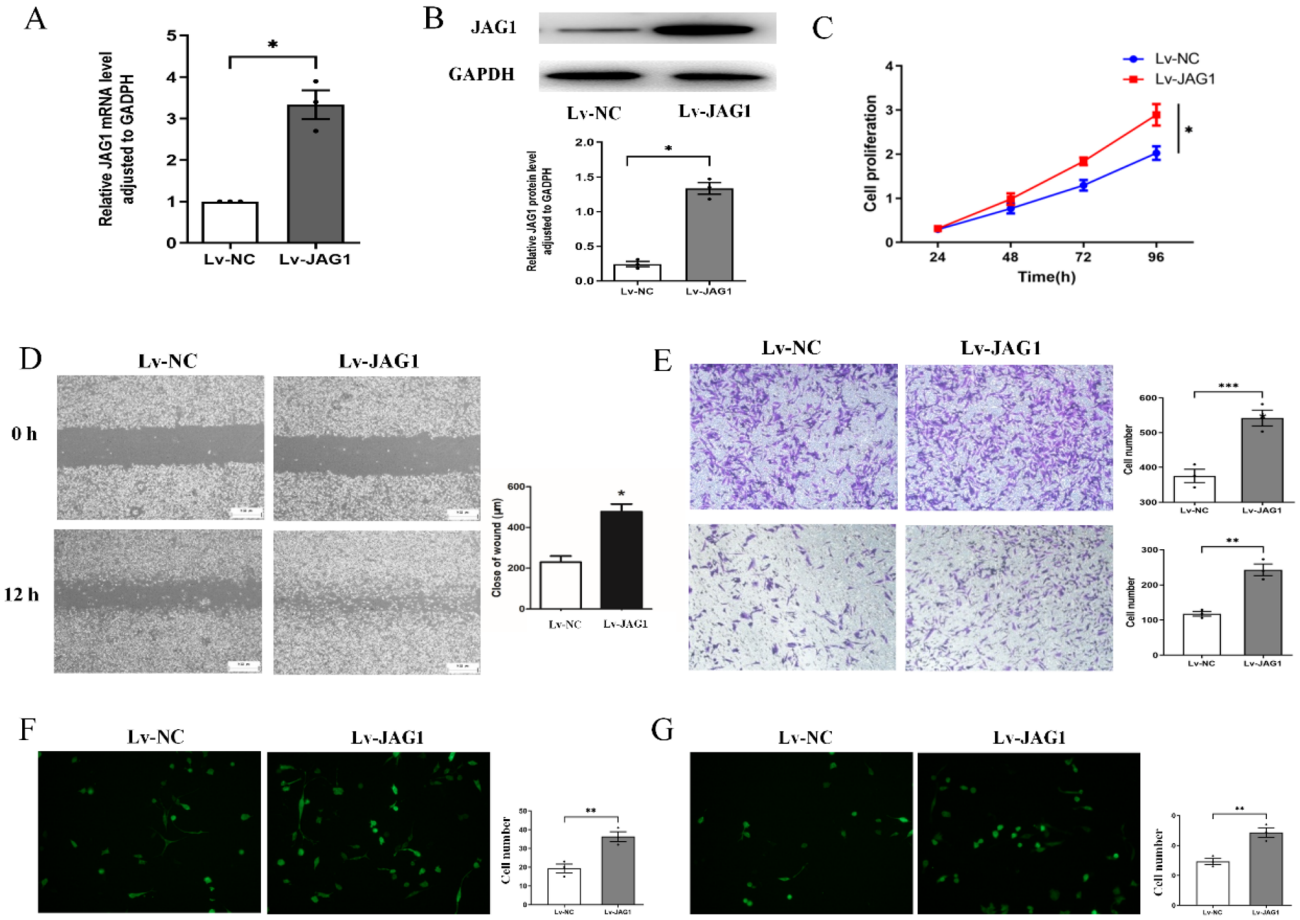
The influence of JAG1 overexpression on BC cells. (**A**) The JAG1 mRNA was explored by qPCR after JAG1 overexpression; (**B**) JAG1 protein was detected by Western Blot after JAG1 overexpression; (**C**) the proliferation of BC cells was assessed by CCK-8 assay; (**D**) cell motility after JAG1 upregulation was detected by wound healing assay; (**E**) transwell assay was conducted to detect the migration (upper panels) and invasion (lower panels) of MDA-MB-231; BBB model was constructed to detect the adhesion (**F**) and penetration (**G**) abilities to BBB.

Subsequently, we upregulated JAG1 in the MDA-MB-231 (Fig. 4A, B). CCK-8 assay demonstrated that JAG1 overexpression significantly promoted BC cell proliferation (Fig. 4C). Wound healing assays confirmed that JAG1 upregulation facilitated the motility of MDA-MB-231 cells (Fig. 4D). Transwell assays confirmed that high JAG1 expression contributed to enhanced cell migration (Fig. 4E, upper panels) and invasion (Fig. 4E, lower panels). Furthermore, the BBB model showed that JAG1 upregulation significantly increased tumor cell adhesion and penetration into the BBB (Fig. 4F, G).

## Discussion

JAG1 plays a carcinogenic role in many types of malignant tumors. Kunanopparat et al.^19^ demonstrated that JAG1 was overexpressed in hepatocellular carcinoma, and showed a significant connection to age and albumin level. In gastric cancer, JAG1 expression in tissues was associated with Borrmann type, and patients with high JAG leve had poorer survival than others^20^. Qiu et al.^21^ confirmed that JAG1 in glioblastoma tissues was significantly increased than non-neoplastic tissues. In addition, high JAG1 expression was connected to advanced clinical features such as the Karnofsky performance scale and symptom duration, as well as poor survival^21^. Zohny et al.^18^ detected JAG1 protein in 90 BC tissues and 42 benign lesions and found the positive rate of JAG1 in BC was higher than that in benign lesions (73.33% vs 26.19%). This research detected JAG1 expression in 200 BC tissues and 47 para-cancer breast tissues and proved that JAG1 protein was highly expressed in BC tissues compared to adjacent non-cancerous tissue (67.5% vs 23.4%), which was consistent with the literature. Correlation analysis confirmed that high JAG1 expression was significantly correlated to advanced lymph node metastasis, distant metastasis, and the TNM stage. In addition, BC patients with high JAG1 expression had a poorer OS than other patients, and JAG1 expression was an independent biomarker for BC patients.

The JAG1-Notch pathway facilitates tumor metastasis via various carcinogenic mechanisms^22–24^. Cohen et al.^25^ found that cyclin D1 is a downstream transcription target of the JAG1-Notch pathway, and knockdown of the JAG1 gene decreases cyclin D1 level, arresting the cell cycle in the G1 phase. In basal-like subtypes of BC, high JAG1 expression induced by the NF-KB pathway could activate the Notch pathway, and then accelerate self-renewal and replication of tumor stem cells^26^. Epithelial-mesenchymal transition (EMT) is essential for BC cells to break through the basement membrane^27^. JAG1-Notch pathway activation facilitates EMT of BC cells by upregulation of the transcriptional suppressor Slug and inhibition of E-cadherin^28^. JAG1 promotes urokinase-type plasminogen activator and enhances the invasive ability of BC cells, resulting in disease progression^29^. Our study showed that JAG1 knockdown in MDA-MB-231 cells inhibited their proliferation, migration, and invasion, whereas JAG1 overexpression had the opposite effects. The above phenomena indicate that JAG1 is a major driving factor promoting the malignant behavior of BC cells.

The BBB is a tightly connected structure formed by cerebrovascular endothelial cells, basement membrane, and astrocytes^30^. It protects the brain from foreign macromolecules and microorganisms, and prevents most chemotherapeutic drugs and antibodies from entering the brain^31^. However, it cannot prevent the invasion of circulating metastatic cells, resulting in unsatisfactory therapeutic effects on brain metastasis. Therefore, it is urgent and necessary to further explore the molecular mechanism of BC cells crossing the BBB. Our results confirmed that JAG1 downregulation decreased the adhesion and penetration of BC cells into the BBB, and high JAG1 resulted in the opposite trend. This finding suggests the vital role of JAG1 in brain metastasis of BC.

In conclusion, we verified that JAG1 is overexpressed in BC tissues, and JAG1 is an independent prognosis biomarker for BC patients. JAG1 promotes BC progression and brain metastasis. This research affords a strong experimental basis for JAG1 to become a therapeutic target for advanced BC patients.

## Methods

All experimental protocols were approved by Inner Mongolia Medical University. All methods were carried out in accordance with relevant guidelines and regulations.

### Tissues and clinical data

Tissue samples analyzed in the research were collected from patients who accepted BC resection in Bayannur Hospital from January 2010 to December 2013. All patients did not receive any cancer treatments such as chemotherapy, radiotherapy, endocrine therapy, targeted therapy, or immune therapy. A total of 200 BC tissues and 47 para-cancer breast tissues were collected. Comprehensive clinicopathological information and follow-up data of patients were also collected. This research gained approval from the Human Research Ethics Committee of Bayannur Hospital.

### Immunohistochemistry (IHC)

IHC was performed according to Envision two-step method. The microarray was dewaxed by xylene and hydrated by graded alcohol. Tris/EDTA solution (PH = 9.0) was selected as the antigen repair buffer, and hot antigen repair was carried out in a pressure cooker using the high-pressure steam method. The tissue microarray was soaked with 3% H_2_O_2_ solution for 10 min and then incubated with 10% goat serum for 1 h. The tissue chips and rabbit anti-human JAG1 antibody (1:200, # ab85763, Abcam, USA) were incubated overnight at 4 °C. The tissue chip was incubated with the goat anti-rabbit antibody (1:500, #ab6721, Abcam, USA) at room temperature for 30 min. DAB dye was used for visualization processing, and the tissue chip was counterstained with hematoxylin.

The expression of JAG1 protein was assessed semi-quantitatively through the combination of staining positive intensity and the proportion of staining positive cells^32^. The IHC cut-off value was 90 via the X-tile software^33^. A score of less than 90 was low or no expression, and the score higher or equal to 90 was a high expression.

### Cell culture and transfection

Cells were maintained in DMEM medium (Invitrogen, Thermo, USA) with 10% fetal calf serum (Invitrogen, Thermo, USA), penicillin (100 IU/mL), and streptomycin (100 µg/mL) at 37 °C, 5% CO_2_. Lentiviral short hairpin RNA-JAG1 (lv-sh-JAG1), lentiviral shRNA-negative control (lv-sh-NC), lentiviral JAG1 overexpression vector (lv-JAG1), and lentiviral JAG1 empty lentiviral vector control (lv-NC) were acquired from GeneCopoeia, Inc. (China).

### Quantitative real-time polymerase chain reaction (qRT-PCR)

TRIzol reagent (Invitrogen, Thermo, USA) was utilized to leach the total RNA of each cell group, and the RNA samples were reversely transcribed into cDNA using the BeyoRT II cDNA synthesis kit (#D7170S, Beyotime, China). Samples were amplified using BeyoFast Probe qPCR Mix (#D7273, Beyotime, China) on an ABI 7500 real-time fluorescent quantitative PCR system. The reference gene was GAPDH, and the relative expression of JAG1 was calculated by the 2^−△△Ct^ method.

### Western blot

RIPA reagent was used to lysate cells and leach total protein. The same amounts of protein samples were separated in SDS-PAGE and then transferred to the PVDF membrane. The membrane was soaked in 5% skim milk at room temperature for 2 h. Rabbit anti-human JAG1 monoclonal antibody (1:1000, # ab85763, Abcam, USA) or GAPDH monoclonal antibody (1:2000, # ab9485, Abcam, USA) was incubated at 4 °C overnight. Finally, the membrane was incubated with a secondary antibody (1:5000, #ab6721, Abcam, USA). The proteins were exposed and photographed in a ChemiDoc XRS^+^ system.

### CCK-8 assay

Cells in each group were added into 96-well plates (2 × 10^4^/well). Subsequently, the cells were cocultured with the mixture of CCK-8 reagent and DMEM medium (1:9) at 24, 48, 72, and 96 h, respectively. The absorbance of each well at 450 nm was measured.

### Wound healing assay

The cells of each group were sown on 6-well plates (5 × 10^5^ cells/well). When the cells converged 60–70% of the well, the original medium was replaced by serum-free DMEM medium. A sterile tip was used to scratch the cell surface. The cell wounds were photographed at 0 h, 12 h, and 24 h.

### Transwell assays

#### Migration assay

Cells in each group were planted in a Transwell chamber (2 × 10^4^/well) with 200 µL DMEM medium and the Transwell chambers were placed in the 24-well plate with 800 µL medium (20% FBS) in each well. The residual cells in the chamber were wiped 24 h later, and the chambers were soaked in methanol for 30 min, and 0.1% crystal violet solution for 20 min. The cells on the lower surface of the membrane were counted.

#### Invasion assay

Matrigel basement membrane matrix (#356234, BD Biosciences, USA) was diluted with serum-free DMEM medium at 4 °C, and then spread in the chamber. The next steps were shown in the migration assay.

### BBB model in vitro

Matrigel basement membrane matrix (#356234, BD Biosciences, USA) was diluted and then was spread on the surface of the upper chamber. Human microvascular endothelial cell (HBMEC) was cultured in each chamber (2 × 10^5^/chamber). When the HBMEC formed a monolayer, the cells in each group were planted in the chamber (2 × 10^4^/chamber) with 200 µL serum-free DMEM medium. The cells on the upper and lower surface of chamber membrane were detected by fluorescence microscope for adhesion and penetration (Supplementary Figures).

### Statistical analysis

All data analysis of this study was carried out by SPSS 22.0 software. The measurement data were expressed as mean ± standard deviation. T test was used for the difference between the two groups in accordance with normal distribution, or the M-W-U test was used. The χ^2^ test was used to compare the rates between groups. Kaplan–Meier method and Log-rank method were used to calculate and compare the survival rate of patients in different groups. Univariate and multivariate Cox regression models were used to analyze the risk factors affecting the prognosis and survival of patients with BC, and *P* < 0.05 was considered statistically significant.

### Ethics approval and consent to participate

This study acquired the approval of the Human Research Ethics Committee of Bayannur Hospital.

### Informed consent

Informed consent was obtained from all the patients participating in the study.

## Supplementary Information

Supplementary Figures.

## Data Availability

The data in this study are available from the corresponding author.
