# Supplementary figures and images for: JAG1 is associated with the prognosis and metastasis in breast cancer
Source: Sci Rep. 2022 Dec 20;12:21986. doi: 10.1038/s41598-022-26241-8 (PMC9768120; doi:10.1038/s41598-022-26241-8)

Figure 1G

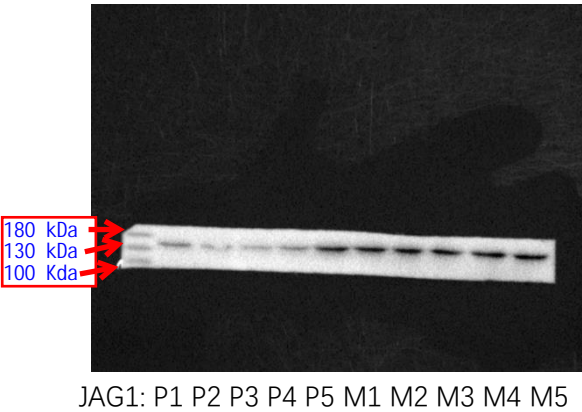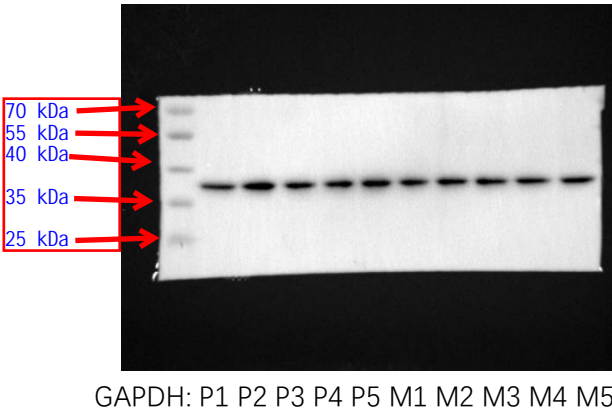

Figure 3B

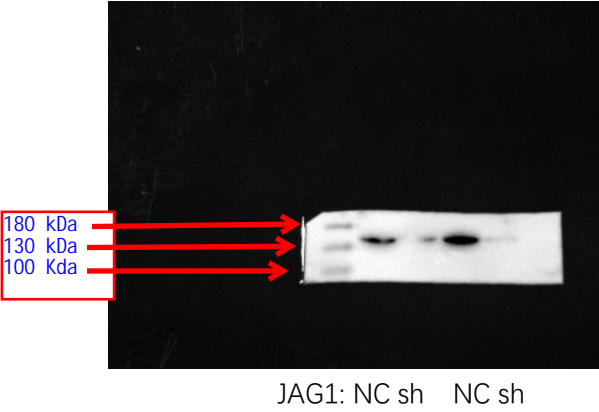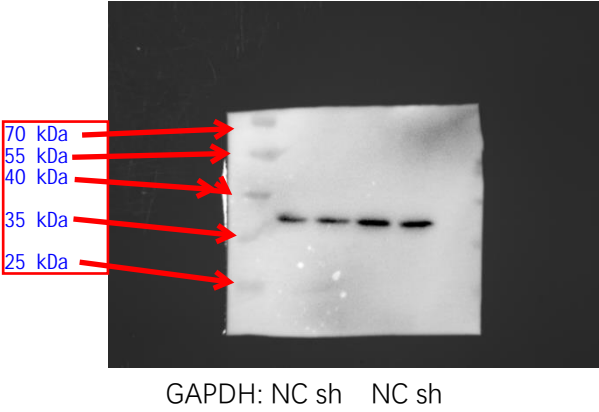

Figure 4B

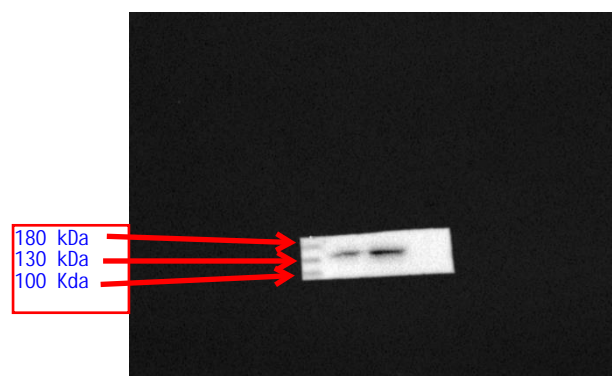

JAG1: NC OE

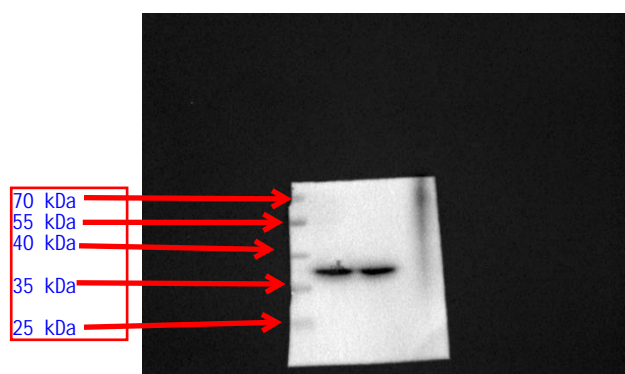

GAPDH: NC OE

Supplement: Supplementary file 1 — Supplementary Figures. [file 41598_2022_26241_MOESM1_ESM.pdf]
